# Exogenous fatty acids alter phospholipid composition, membrane permeability, capacity for biofilm formation, and antimicrobial peptide susceptibility in *Klebsiella pneumoniae*

**DOI:** 10.1002/mbo3.635

**Published:** 2018-04-27

**Authors:** Chelsea R. Hobby, Joshua L. Herndon, Colton A. Morrow, Rachel E. Peters, Steven J. K. Symes, David K. Giles

**Affiliations:** ^1^ Department of Biology, Geology, and Environmental Science The University of Tennessee at Chattanooga Chattanooga TN USA; ^2^ Department of Chemistry and Physics The University of Tennessee at Chattanooga Chattanooga TN USA

**Keywords:** biofilms, fatty acids, *Klebsiella pneumoniae*, phospholipids, polymyxin B

## Abstract

*Klebsiella pneumoniae* represents a major threat to human health due to a combination of its nosocomial emergence and a propensity for acquiring antibiotic resistance. Dissemination of the bacteria from its native intestinal location creates severe, complicated infections that are particularly problematic in healthcare settings. Thus, there is an urgency for identifying novel treatment regimens as the incidence of highly antibiotic‐resistant bacteria rises. Recent findings have highlighted the ability of some Gram‐negative bacteria to utilize exogenous fatty acids in ways that modify membrane phospholipids and influence virulence phenotypes, such as biofilm formation and antibiotic resistance. This study explores the ability of *K. pneumoniae* to assimilate and respond to exogenous fatty acids. The combination of thin‐layer chromatography liquid chromatography‐mass spectrometry confirmed adoption of numerous exogenous polyunsaturated fatty acids (PUFAs) into the phospholipid species of *K. pneumoniae*. Membrane permeability was variably affected as determined by two dye uptake assays. Furthermore, the availability of many PUFAs lowered the MICs to the antimicrobial peptides polymyxin B and colistin. Biofilm formation was significantly affected depending upon the supplemented fatty acid.

## 1 INTRODUCTION

The advent of multidrug‐resistant bacteria has significantly hampered advances in treatment. Among the most concerning emerging resistant opportunistic pathogens is *Klebsiella pneumoniae*, a member of the notorious “ESKAPE bugs” (Rice, 2009). *K. pneumoniae*, a normal inhabitant of the human gut, has recently gained resistance to carbapenems, a last line of defense for treatment of healthcare‐associated infections. The difficulty in managing and treating *K. pneumoniae* infections stems from its very origin, from within a compromised host located in close proximity to many other susceptible hosts. Surfacing in China nearly 5 years ago, the emergence of cabapenem‐resistant *K. pneumoniae* (CRKP) and other hypervirulent, antibiotic‐resistant and persistent strains have established this bacteria as a significant threat to the health of hospital patients (Liu, Li, & Zhang, 2014; Shon, Bajwa, & Russo, 2013; Zhang, Zhao, & Wang, 2016).

Recent studies from our laboratory have explored the various contributions of exogenous fatty acids on the physiology of Gram‐negative pathogens (Eder et al., 2017; Moravec et al., 2017). In addition to altering the membrane phospholipid constitution, notable changes to the MICs of antimicrobial peptides were observed, hinting toward potential therapeutic value. This study seeks to explore whether *K. pneumoniae* also responds to exogenous fatty acids. As in *E. coli*, the process of fatty acid uptake and degradation involves external recognition and transport of the fatty acid through the outer membrane, a process mediated by FadL (Nunn & Simons, 1978). The subsequent passage through the periplasm and inner membrane in not completely understood, but fatty acids are activated by FadD on the cytosolic face of the inner membrane, producing acyl‐coenzyme A. Fatty acid fates include degradation via B‐oxidation or recycling of the fatty acid directly into membrane phospholipid assembly with the aid of acyltransferases (Yao & Rock, 2017; Zhang & Rock, 2008a, 2008b). Many other reports have described a variety of bacterial responses to exogenous fatty acids, suggesting that these molecules may play roles in recognizing and responding to the environment (Golubeva, Ellermeier, Cott Chubiz, & Slauch, 2016; Lai et al., 2005; Liaw, Lai, & Wang, 2004; Shleeva et al., 2013). The aim of this study is to examine whether *K. pneumoniae* has the capacity for exogenous fatty acid assimilation into membrane phospholipids, as well as a survey of phenotypic responses to gauge the potential relevance of exogenous fatty acids in survival, persistence, and virulence.

The exogenous fatty acid response of *K. pneumoniae* has not been studied previously. To assess the incorporation of exogenous fatty acids, extracted bacterial phospholipids were analyzed by thin‐layer chromatography and UPLC/ESI‐MS. We demonstrate the remodeling of membrane phospholipids as evidenced by the assimilation of supplemented fatty acids into extracted phospholipids. All fatty acid tested enhanced growth in minimal media and supported growth when administered as a sole carbon source. Assays using crystal violet and ethidium bromide indicated changes to membrane permeability, while biofilm formation was also influenced by several fatty acids. A key finding was the measured difference in MIC for two antimicrobial peptides when fatty acids are available.

## 2 MATERIALS AND METHODS

### 2.1 Bacterial strains and growth conditions

*Klebsiella pneumoniae* ATCC13883 was used in this study. CM9 and G56 minimal media (0.4% Glucose, 0.4% casamino acids (Fisher BioReagents), supplemented with 150 mmol/L NaCl) were used for growth of bacteria in experiments, except for sole carbon source experiments which were performed in M9 minimal media lacking glucose. All experiments were performed at 37°C. Fatty acids used in this study were purchased from Cayman Chemicals [linoleic acid (18:2), α‐linolenic acid (18:3α), γ‐linolenic acid (18:3γ), dihomo‐γ‐linolenic acid (20:3), arachidonic acid (20:4), eicosapentaenoic acid (20:5), and docosahexaenoic acid (22:6)] and administered at a concentration of 300 μmol/L for each experiment, except for sole carbon source where they were administered at 1 mmol/L.

### 2.2 Bacterial lipid extraction and thin‐layer chromatography

Lipids were extracted from 14 ml of bacterial culture by the method of Bligh and Dyer (1957) and spotted onto Silica Gel 60 TLC plates. Lipids were separated using a solvent system consisting of chloroform, methanol and acetic acid (65:25:10 v/v/v). Once dry, the plates were sprayed with a solution of 10% sulfuric acid in 100% ethanol and exposed to 150°C for approximately 1 m. The plates were scanned using a Canon CanoScan 9000F.

### 2.3 Ultra performance liquid chromatography/ESI‐mass spectrometry

Lipids were extracted from 20 ml of bacterial culture grown in G56 minimal media in the presence or absence of 300 μmol/L of each PUFA. Prior to analysis, dried lipid extracts were massed and then brought up in sufficient diluent to produce a 400 ppm (total lipid) sample. Diluent consisted of a 50:50 mixture of solvents A and B where A = 30:70 25 mmol/L pH 6.7 ammonium acetate:methanol and B = methanol. All reagents were Optima grade (Fisher Scientific) and 18.2 MΩ cm water was produced from a Direct Q3 Milli‐Q system (Millipore, Bedford, MA). Samples were prepared in LC‐MS certified glass autosampler vials and 5 μl was injected for analysis. Chromatographic separation was achieved using gradient elution on an ACQUITY UPLC system (Waters, Milford, MA) equipped with a BEH C8 column (2.1 × 100 mm; 1.7 μm particles). The binary LC gradient began with a 2 min hold at 50% B, followed by a linear ramp to 100% B by 10 min, followed by a return to initial conditions for 1 min. Because of the cycle time of the autosampler, each sample experienced 3 min of re‐equilibration at initial conditions before analysis. A methanol blank was injected after every 3 samples and showed no detectable signals indicating there is no carryover from previous injections. Detection was by quadrupole mass spectrometry in full scan mode (*m/z* 200 – 800 with 0.500 s scan time) following electrospray ionization in the negative mode. The Quattro Micro (Micromass UK Ltd.) mass spectrometer was controlled by MassLynx v4.1 software. Mass spectrometry conditions (1.5 kV capillary; 50 V cone; 350°C desolvation temperature) allowed simultaneous observation of [M – H]^−^ parent ions and their cone fragments (i.e., in‐source fragmentation) in the mass spectra providing confidence in the phospholipid assignments. The most prominent cone fragments consist of (R_x_CO_2_)^−^ ions where *x* = 1, 2 corresponding to sn‐1 and sn‐2 positions on the glycerol backbone. For our purposes, which is to show fatty acid incorporation in the phospholipid membrane, the exact positions of the carboxylate substituents is not critical, only that one of the chains (or both) consist of the fatty acid that was supplemented.

### 2.4 Crystal violet uptake assay

*K. pneumoniae* was grown in 7 ml of G56 minimal media in the presence and absence of 300 μmol/L of each PUFA to logarithmic phase (all cultures were captured at an OD_600_ of 0.85–0.95). The cultures were gently pelleted (2,500 × g, 10 m), washed with phosphate buffered saline (PBS) and resuspended in 5 ml PBS at an OD of 0.4. Crystal violet (5 μg/ml) was added to the cells and cultures were gently agitated (50 rpm). Every 5 m, 1 ml was removed, pelleted, and the supernatant was assessed spectrophotometrically at 590 nm. Inclusion of a control (containing CV but no bacteria) allowed normalization of the data. The amount of CV measured (representing dye not taken up) was converted to percentage of uptake using Excel. Three biological replicates were performed and all standard deviations were calculated to be less than 5% (not graphed for visual clarity). Statistical significance was determined by a Student's *t* test (paired, two‐tailed, *p* < .02).

### 2.5 Ethidium bromide uptake assay

*K. pneumoniae* was grown in 7 ml of CM9 minimal media in the presence and absence of 300 μmol/L of each PUFA to logarithmic phase (all cultures were captured at an OD_600_ of 0.85‐0.95). The cultures were gently pelleted (2,500 × g, 10 m), washed with phosphate buffered saline (PBS) and resuspended in 5 ml PBS at an OD of 0.7. Ethidium bromide (5 μg/ml) was added to the cells and cultures were gently agitated (50 rpm). Every 5 m, 1 ml was removed, pelleted, and the supernatant was assessed fluorometrically using an excitation wavelength of 530 nm and detection wavelength of 585 nm. Inclusion of a control (containing EtBr but no bacteria) allowed normalization of the data and represents the maximal fluorescence intensity (measured as 420 nm and plotted as the “zero” time for each sample). The measurements were performed on a Varian Cary Eclipse Fluorescence Spectrophotometer with a 20‐nm excitation slit width. Three biological replicates were performed and statistical significance was determined by a Student's *t* test (paired, two‐tailed, *p* < .02).

### 2.6 Antimicrobial peptide susceptibility assay

*K. pneumoniae* was grown to exponential phase of growth in G56 minimal media in the presence and absence of individual fatty acids (300 μmol/L). Cultures were centrifuged, washed with G56, and resuspended at an OD_600_ of 0.13. Bacterial inoculum (170 μl) was supplemented with 300 μmol/L of each fatty acid and added to twofold concentrations of each antimicrobial peptide (30 μl) [polymyxin B sulfate (Fisher Biosciences); colistin sulfate (Adipogen); imipenem (LKT Laboratories, Inc.)] for a total of 200 μl per well (bacterial starting OD_600_ = 0.1). Plates were incubated for 24 hr at 37°C and absorbance was read at 600 nm using a Biotek Synergy microplate reader. Two independent experiments were performed in triplicate and p‐values were determined using the Student’s *t* test (2‐tailed, paired). A significant change in MIC was considered to be at least a fourfold difference from the control.

### 2.7 Biofilm assay

Assessment of biofilm formation was performed using the protocol by O'Toole (2011). Briefly, *K. pneumoniae* was grown overnight in Luria broth and cultures were prepared in 96‐well microtiter plates containing G56 or M9 minimal media supplemented with or without each fatty acid. Following incubation for 24 hr at 37°C, planktonic cells were removed and the plates were gently washed three times with dH_2_O. To each well, 125 μl of a 3% crystal violet solution was added and plates were incubated at room temperature for 15 m. Plates were washed 3 times with dH_2_O and allowed to dry at least 3 hr. 125 μl of 30% acetic acid was added and allowed to incubate for 15 m. The solubilized crystal violet was transferred to a fresh microtiter plate and absorbance was read at 550 nm using a Biotek Synergy microplate reader. Two independent experiments were performed in octuplet and p‐values were determined using the Students *t* test (2‐tailed, paired).

### 2.8 Bioinformatics

Table 1 was generated by consulting two databases: the National Center of Biotechnology Information's Basic Local Alignment Search Tool (BLAST) (https://blast.ncbi.nlm.nih.gov/Blast.cgi) and the Department of Energy's Integrated Microbial Genomes (IMG) ‘Find Genes’ tool using the gene product names as queries. Briefly, the protein sequences of *E. coli* MG1655 FadL (b2344), FadD (b1805), phospholipid acyltransferases (b4041, b3018, b1090, b3059) constituted input for homolog searches in the representative sequenced *Klebsiella* species. The Expect threshold was set to 1e‐10. Only homologs identified by BLAST with a Max Score greater than 100 were included. All locus tags were identified using BLAST and/or IMG.

**Table 1:** *Klebsiella* species possesses an expanded repertoire of genes involved in fatty acid uptake

	FadL	FadD	PlsB/C/X/Y
*K. pneumoniae subsp. pneumoniae* HS11286	KPHS_37950KPHS_37960	KPHS_33350KPHS_26370KPHS_43090 (aas)	KPHS_02640 (plsB)KPHS_45630 (plsC)KPHS_19670 (plsX)KPHS_45920 (plsY)
*K. oxytoca* KONIH1	KONIH1_20880	KONIH1_17840KONIH1_24445KONIH1_24095 (aas)	KONIH1_01795 (plsB)KONIH1_25455 (plsC)KONIH1_11290 (plsX)KONIH1_25620 (plsY)
*K. aerogenes* KCTC 2190	EAE_24680EAE_14945	EAE_22550EAE_16515EAE_02180 (aas)	EAE_08420 (plsB)EAE_03525 (plsC)EAE_16360 (plsX)EAE_03665 (plsY)
*K. variicola* At‐22	Kvar_1323	Kvar_1867Kvar_3252Kvar_0825 (aas)	Kvar_4819 (plsB)Kvar_0660 (plsC)Kvar_3292 (plsX)Kvar_0632 (plsY)

*Klebsiella* species were bioinformatically assessed (see Methods) for homologs to long chain fatty acid transporter (FadL), long chain fatty acyl‐CoA synthetase (FadD) and acyltransferases (PlsB/C/X/Y) involved in acquisition and assimilation of exogenous fatty acids. The identification of putative homologs supports the capability of *Klebsiella* species to acquire and utilize exogenous fatty acids.

## 3 RESULTS

### 3.1 Growth characteristics of *K. pneumoniae* in the presence of exogenous PUFAs

**Figure 1:**
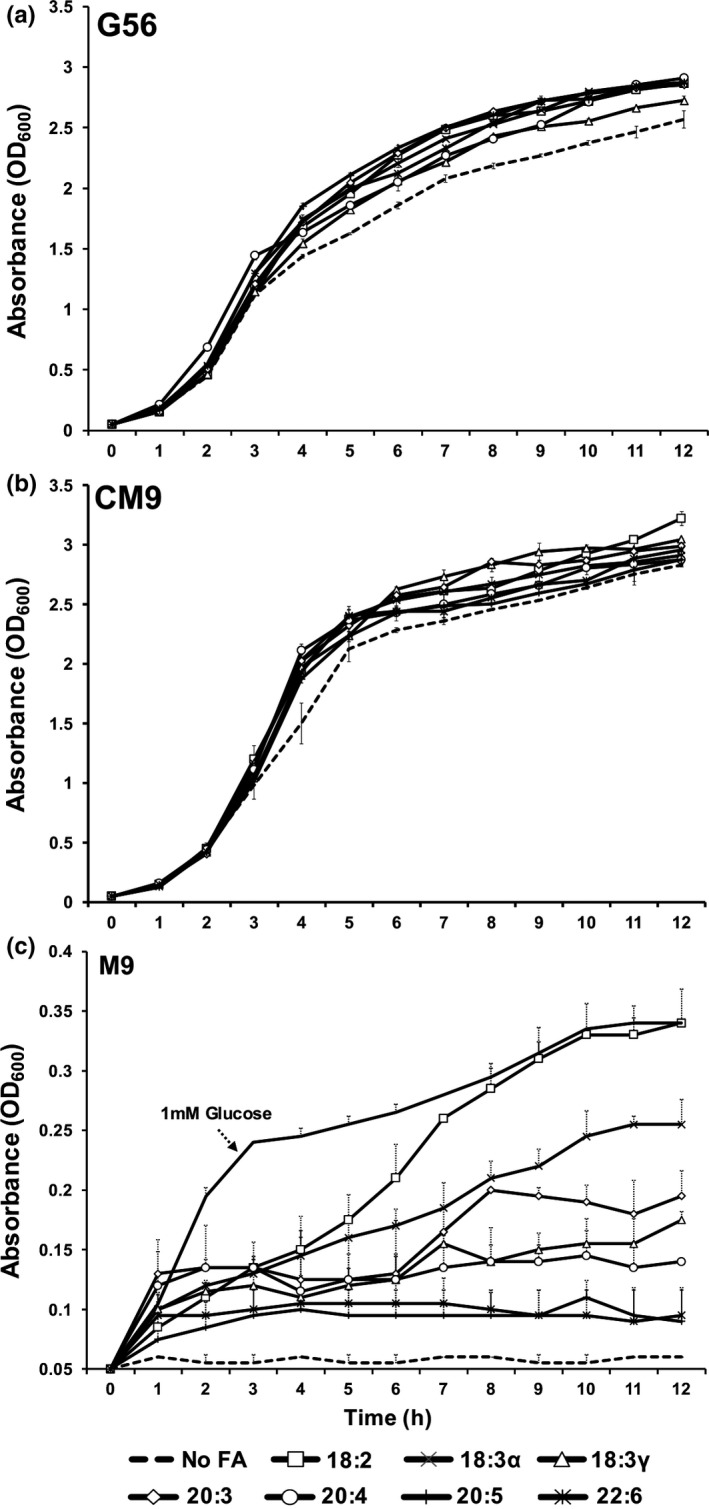
Growth characteristics of *Klebsiella pneumoniae* in the presence of exogenous fatty acids. (a & b) Bacteria were grown with 300 μmol/L fatty acids (linoleic acid [18:2], alpha‐linoleic acid [18:3α], gamma‐linolenic acid [18:3γ], dihomo‐gamma‐linolenic acid [20:3], arachidonic acid [20:4], eicosapentaenoic acid [20:5] and docosahexaenoic acid [22:6]) in G56 and CM9 minimal media (starting OD = 0.05) for 12 hr at 37°C. (c) Exogenous fatty acids (1 mmol/L) were supplied in M9 minimal media (no glucose) as the sole carbon source and growth was monitored for 12 hr. The negative control contained no carbon source, while the positive control contained 1 mmol/L glucose. Each growth curve represents the average of two biological replicates with standard deviations

*K. pneumoniae* causes a broad spectrum of human infections, such as bacteremia, pneumonia, meningitis, and urinary tract infections. Thriving in a plethora of environments necessitates detection of external cues to respond appropriately for survival and persistence. Fatty acids are examples of molecules that could be encountered in their free form or enzymatically cleaved by bacteria or host from larger lipid molecules. The presence of fatty acids has been documented in many tissues that represent infection sites for *K. pneumoniae* (Farrell, Mischler, Engle, Brown, & Lau, 1985; Kim et al., 2003; Patil & Magar, 1960). To examine the effects of fatty acids on growth, a physiologically relevant concentration of fatty acid (300 μmol/L) was supplemented and chosen for all experiments. Bacterial growth was measured using two minimal media supplemented with individual fatty acids. All fatty acids increased growth of *K. pneumoniae* in both minimal media (Figure 1a and b), particularly after 3 hr.

### 3.2 Exogenous PUFAs support *K. pneumoniae* growth as the sole carbon source

When supplemented into minimal media as the sole carbon source, all fatty acids supported growth of *K. pneumoniae* (Figure 1c). Interestingly, higher carbon length and degree of unsaturation directly correlated with decreased growth of bacteria. Linoleic acid (18:2) allowed growth comparable to supplementation with 1 mmol/L glucose.

### 3.3 PUFA exposure results in altered phospholipid profiles of *K. pneumoniae*

**Figure 2:**
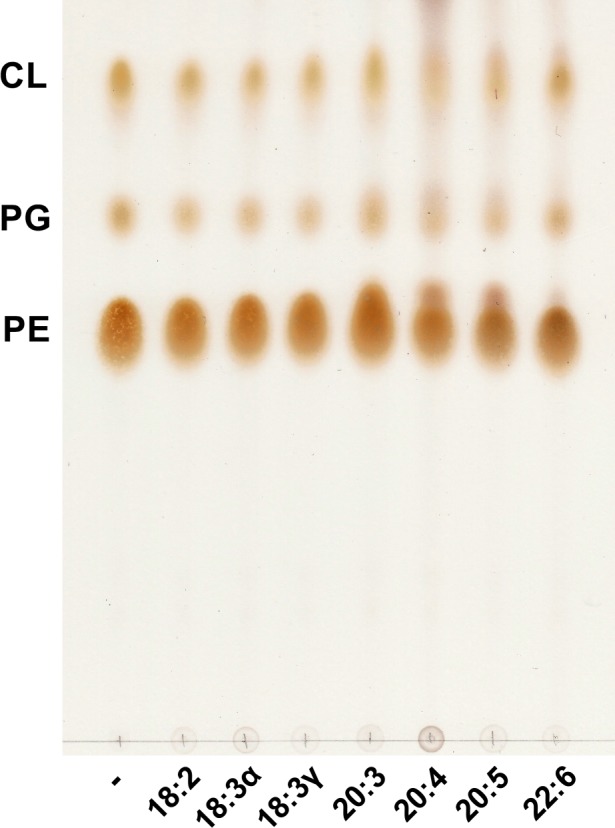
Thin‐layer chromatography of phospholipids extracted from *Klebsiella pneumoniae* grown in the presence of individual polyunsaturated fatty acids. Bacteria were grown to exponential phase (OD ≈ 0.8) in G56 minimal media at 37°C with or without 300 μmol/L of the indicated fatty acids (linoleic acid [18:2], alpha‐linoleic acid [18:3α], gamma‐linolenic acid [18:3γ], dihomo‐gamma‐linolenic acid [20:3], arachidonic acid [20:4], eicosapentaenoic acid [20:5] and docosahexaenoic acid [22:6]) prior to Bligh and Dyer extraction of phospholipids and separation by TLC in the solvent system chloroform/methanol/acetic acid (65:25:10 v/v/v). The plate was charred and scanned to produce the final image

To test the effect of various PUFAs on *K. pneumoniae* phospholipids, bacteria were grown in the presence and absence of micromolar concentrations of each fatty acid. Cultures were grown in minimal medium to exclude any fatty acid contributions of complex media, as are present in Luria and tryptic soy broths. Bacterial phospholipids were extracted and examined by thin‐layer chromatography (TLC). The major phospholipids produced by *K. pneumoniae* include phosphatidylethanolamine (PE), phosphatidylglycerol (PG) and cardiolipin (CL) (Figure 2). The chromatogram revealed some migrational shifts upward as carbon number and unsaturation of the exogenously supplied fatty acid increased, indicative of production of phospholipid species with a higher hydrophobicity compared to the control. The shifts are more evident in PE and in some cases, two distinct species of the same phospholipid can be discerned, particularly as carbon length and unsaturation is increased (see PE w/20:4 and 20:5).

### 3.4 UPLC/ESI‐MS analyses indicate assimilation of exogenous PUFAs into *K. pneumoniae* phospholipids

**Figure 3:**
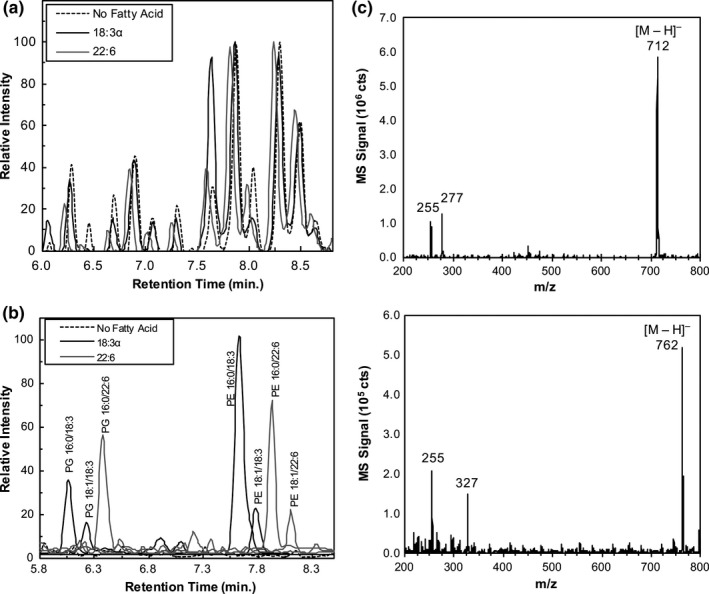
Ultra performance liquid chromatography/mass spectrometry of isolated phospholipids from *Klebsiella pneumoniae* grown in the presence and absence of fatty acids. *K. pneumoniae* was grown with or without 300 μmol/L of a given fatty acid at 30°C in G56 (pH 7.4) to logarithmic phase. Lipids were extracted using the Bligh and Dyer method but included an extra wash step to increase the purity of the isolated lipids. The lipid extract was injected (5 μl) into a Waters UPLC for gradient elution using a reversed phase C‐8 column. [M‐H]^−^ ions were detected by quadrupole mass spectrometry following electrospray ionization. (a) Total ion chromatograms (*m/z* 200 – 800) comparing the control to cultures exposed to either 18:3α or 22:6 fatty acid. All other comparisons are provided in Supplemental Data. Changes in the chromatograms compared to the control suggest modifications of the phospholipid profile that depend on the supplied fatty acid. (b) Extracted ion chromatograms (XIC) showing new peaks, in the exposed samples, that are absent in the control. The labeled peaks are predicted using the Lipid Maps Database (http://www.lipidmaps.org/) and are based on the *m/z* of the parent ion and the presence of cone fragments induced by the ESI conditions. From left to right, the XIC's correspond to the following *m/z* values: 743.5, 769.5, 793.5, 712.5, 738.5, 762.5, 788.5. The control was mass filtered for all of these same ions but only noise was present. As a result, only the XIC at 793.5 is used to represent the control. (c) Example mass spectra showing parent ions and their cone fragments allowing positive identification of PE 16:0/18:3 (7.6 min peak in the XIC; top) and PE 16:0/22:6 (7.9 min peak in the XIC; bottom). Peaks at *m/z* 255, 277, and 327 correspond to cone fragment carboxylate anions (RCOO)^–^ of 16:0, 18:3, and 22:6, respectively

The qualitative changes observed by TLC (Figure 2) were further investigated using ultra performance liquid chromatography/electrospray ionization mass spectrometry (UPLC/ESI‐MS). Bacterial lipids were extracted following growth with or without individual PUFAs and subjected to chromatographic separation using reversed‐phase gradient elution resulting in good separation between PE and PG, the two major phospholipids (PL) synthesized by *K. pneumoniae*. Analyses of 400 ppm (total lipid extract) samples yielded chromatograms reflecting structural changes to the phospholipid profile, dependent upon the specific fatty acid supplemented. Total ion chromatograms (TIC) and extracted ion chromatograms (XIC) from the control (no fatty acid in growth media) and select fatty acid exposed cultures are shown in Figure 3. Only two fatty acid exposed samples are shown in Figure 3 for the sake of clarity, but a more extensive comparison showing all other fatty acid exposed samples can be found in the Supplemental Materials. The appearance of new chromatographic peaks in all fatty acid exposed samples clearly indicates that the phospholipid profiles have been altered compared to the control (Figure 3 and Figure S1). All fatty acids tested resulted in altered phospholipid profiles and these differences depend on the specific fatty acid that was supplemented.

As observed by TLC, the LC‐MS results detected abundant PE and PG species. Mass spectrometry confirms that these phospholipids are composed of fatty acyl chains derived from the exogenously supplied fatty acid. Mass filtering for specific parent ions resulted in XICs showing baseline resolution of closely related phospholipids (Figure 3b). Such resolution allows confident assignment of specific PLs to specific parent ion m/z values. Analysis of the [M‐H]^−^ ions shows that the new peaks correspond to phospholipid species comprised of at least one acyl chain matching the supplied fatty acid. This is most clearly shown in the extracted ion chromatograms in Figure 3b (mass spectra for each of these peaks can be found in Figure S2). For example, phospholipids from fatty‐acid supplemented cultures had peaks corresponding to [M‐H]^−^ ions of *m/z* 743.5 (6.1 min), 769.5 (6.2 min), and 793.5 (6.4 min), which are absent from the control. These ions correspond to PG species with fatty acyl compositions of 16:0/18:3, 18:1/18:3, and 16:0/22:6, respectively. Likewise, PE species also show modification with XIC peaks at *m/z* 712.5 (7.6 min), 738.5 (7.8 min), 762.5 (7.9 min), and 788.5 (8.1 min) corresponding to PE 16:0/18:3, PE 18:1/18:3, PE 16:0/22:6, and PE 18:1/22:6 (Figure 3b). Analysis of parent ions and their cone fragments confirmed the fatty acid constitution for each phospholipid mass spectra (e.g., Figure 3c). Although all data is not shown, all fatty acids tested resulted in similar phospholipid tail alterations corresponding to phospholipid species comprised of at least one acyl chain matching the supplied fatty acid. These results indicate that both PG and PE phospholipids show incorporation of every exogenously supplied fatty acid tested.

### 3.5 Exogenous PUFAs affect hydrophobic compound uptake in *K. pneumoniae*

**Figure 4:**
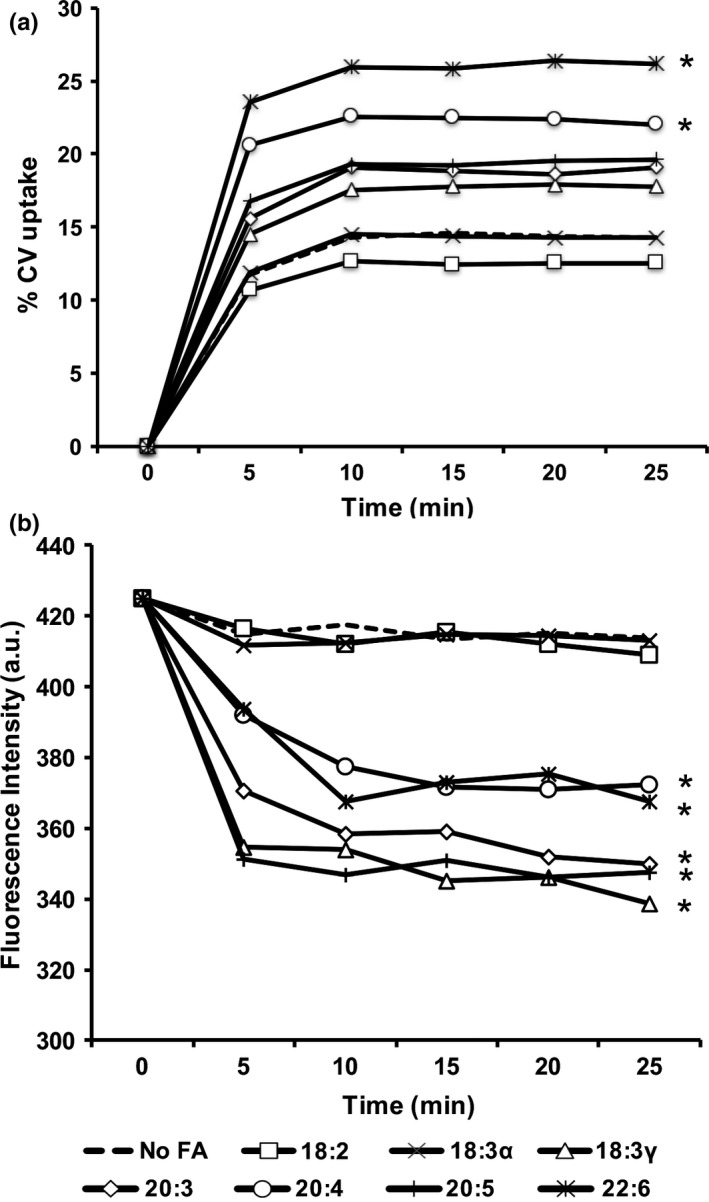
The effect of exogenous fatty acids on permeability in *Klebsiella pneumoniae*. (a) Bacteria were grown at 37°C in G56 (pH 7.4) with and without 300 μmol/L of the indicated fatty acids to mid‐log phase (OD = 0.8). Cultures were gently pelleted, washed with PBS and resuspended in an equal volume of PBS (OD_600_ = 0.7). The amount of CV in the supernatant following centrifugation was measured at regular intervals and expressed graphically as percentage of CV uptake. Results represent means of three independent determinations of CV uptake. All standard deviations were less than 3% (not graphed for visual clarity) and asterisks indicate significant difference (*,*p* < .002) as compared to control. (b) Bacteria were grown at 37°C in CM9 with and without 300 μmol/L of the indicated fatty acids to mid‐log phase (OD = 0.8). Cultures were gently pelleted, washed with PBS and resuspended in an equal volume of PBS (OD_600_ = 0.7). The amount of EtBr in the supernatant following centrifugation was measured as fluorescence emission intensity at 585 nm (excitation wavelength of 530 nm)

The observed structural modifications to phospholipids led to the hypothesis that membrane permeability was affected. By measuring bacterial uptake of the hydrophobic compound crystal violet, it was evident that the fatty acids variably altered permeability in *K. pneumoniae* (Figure 4). Depending upon fatty acid supplemented, the amount of dye uptake was altered. In the crystal violet assay, significant differences (*p* < .02) were observed for two fatty acids that increased permeability (22:6 and 20:4). Measurement of ethidium bromide uptake generally supported the CV uptake results, with five fatty acids exhibiting elevated uptake and the same two eliciting minimal to no change (18:2 and 18:3α). Clearly, exposure to fatty acids impacts permeability in *K. pneumoniae*.

### 3.6 Exogenous PUFAs alter antimicrobial peptide resistance in *K. pneumoniae*

**Figure 5:**
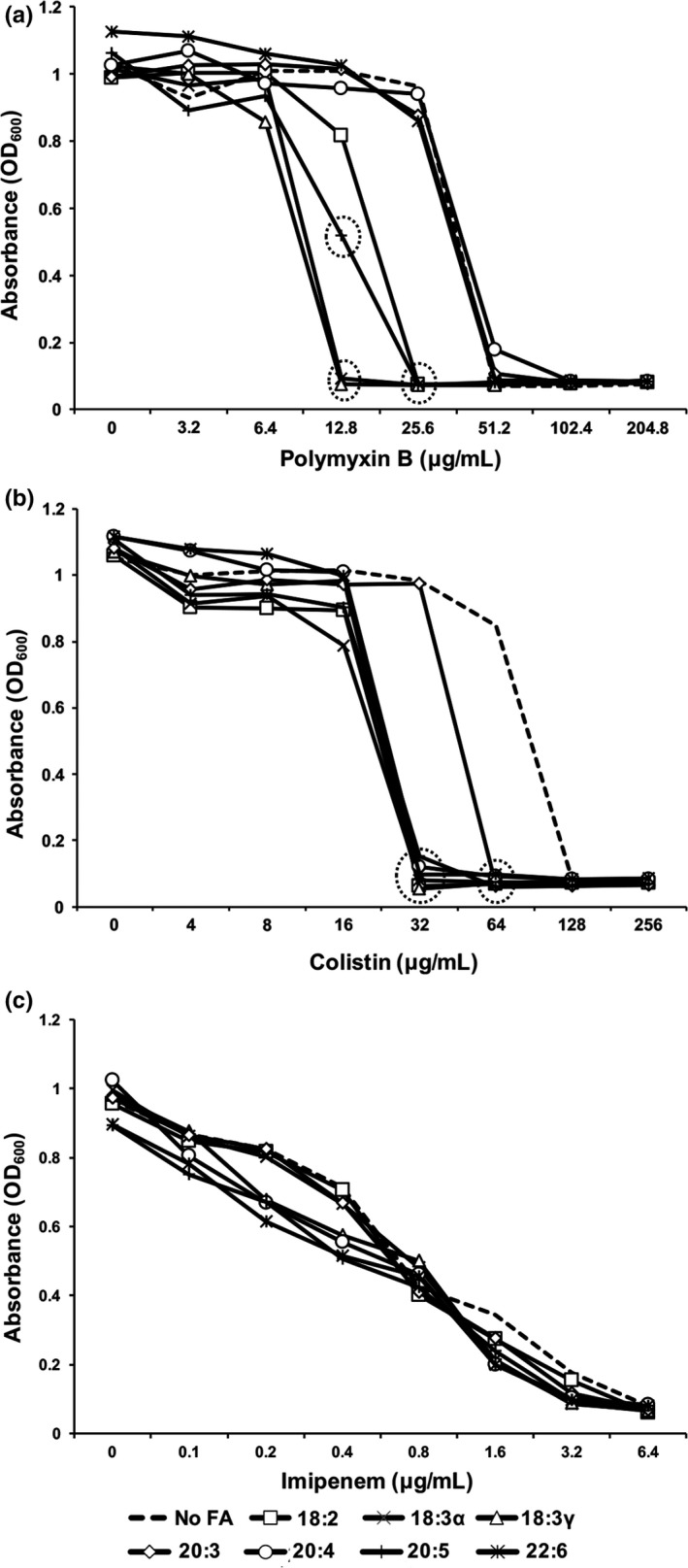
The effect of exogenous fatty acids on polymyxin B, colistin and imipenem resistance in *Klebsiella pneumoniae*. Bacteria were grown at 37°C in G56 (pH 7.4) with and without 300 μmol/L of the indicated fatty acids to mid‐log phase (OD = 0.8). Cultures were pelleted, washed with G56 and resuspended in G56 to an OD_600_ of 0.1. Fatty acids were added to a final concentration of 300 μmol/L. The bacterial suspension was distributed into microtiter plates and two‐fold concentrations of (a) polymyxin B, (b) colistin or (c) imipenem were added. After 24 hr incubation at 37°C, the optical density (600 nm) was read using a Biotek Synergy microplate reader. Experiments were conducted in triplicate, with each value representing the mean. Most standard deviations (<0.04) are masked by the symbol markers and have been omitted for clarity. Symbols circled by dotted line indicate significant differences (*p* < .002) as compared to the control (no fatty acid) at the particular antimicrobial concentration. Significant changes to MIC were considered for differences in at least fourfold compared to the control grown without fatty acid supplementation

It was hypothesized that the assimilation of exogenous fatty acids would affect bacterial response to some antimicrobials, particularly antimicrobial peptides that weaken bacterial membranes by inserting themselves into the bilayer. The impacts of fatty acids on polymyxin B, colistin and imipenem resistance were investigated using microtiter plate broth dilution MIC assays (Figure 5). *K. pneumoniae* exhibited increased susceptibility to polymyxin B and colistin following growth with fatty acids (Figure 5a and b). Both linolenic acids elicited the most vulnerability to polymyxin B, while linoleic acid (18:2) and eicosapentaenoic acid (20:5) also reduced the MIC. All fatty acids except dihomo‐γ‐linolenic acid (20:3) lowered the MIC of colistin by fourfold. Susceptibility to the beta‐lactam antibiotic imipenem was not affected by supplementation with fatty acids (Figure 5c).

### 3.7 PUFAs impact biofilm formation in *K. pneumoniae*

**Figure 6:**
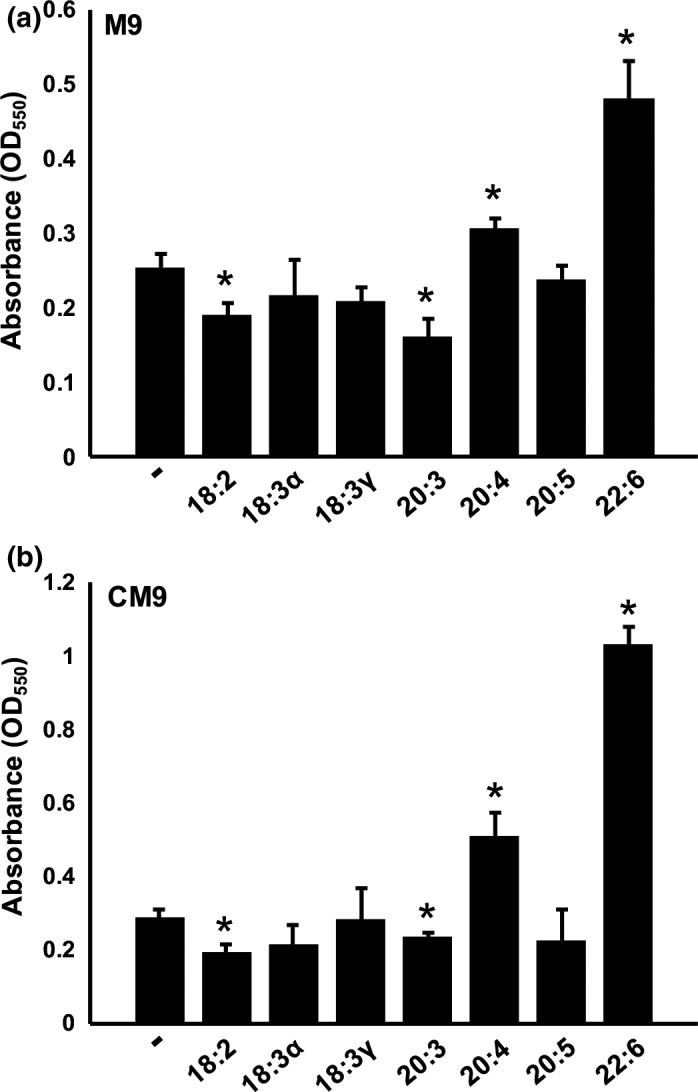
Incubation with exogenous fatty acids alters biofilm formation in *Klebsiella pneumoniae*. Overnight cultures were pelleted, washed, resuspended in the appropriate media and inoculated onto microtiter plates (starting OD ~0.1) in octuplet. Each culture was grown in the presence of 300 μmol/L of the indicated fatty acids. After 96 hr incubation, the biofilm assay by O'Toole was performed in M9 (a) and CM9 (b) minimal media. The absorbance (OD_590_) was measured using a Biotek Synergy microplate reader. Values represents the mean (± SD) of two independent experiments performed in octuplet. Significant differences in biofilm formation were observed as determined by Student's *t* test and are indicated by asterisks (*,*p *<* *.005)

Biofilms are well‐established as bacterial microcolonies that facilitate survival and persistence, as well as contribute to virulence during infection. The effects of fatty acids on biofilm formation was assessed using a crystal violet assay. The assay was performed in M9 minimal media supplemented with and without casamino acids. Regardless of casamino acid supplementation, the same four fatty acids elicited a significant effect on biofilm formation. (Figure 6). The presence of linoleic acid (18:2) and dihomo‐γ‐linolenic acid (20:3) lowered biofilm formation, whereas arachidonic acid (20:4) and docosahexaenoic acid (22:6) led to increased biofilm formation. The elevated amounts of biofilm in CM9 are attributed to the casamino acids, allowing more optimal growth conditions and accounting for the higher overall biofilm measurements.

## 4 DISCUSSION

The experiments in this study have explored the influence of exogenous fatty acids on the membrane phospholipid constitution and behavior of *K. pneumoniae*. Fatty acids supplied during growth were identified by UPLC/ESI‐MS as structural components of bacterial phospholipids, and these alterations were phenotypically assessed to gauge the impact on bacterial survival and resistance to stress. The exposure to fatty acids also affected growth, membrane permeability, biofilm formation and antimicrobial peptide resistance.

The assimilation of and responses to environmental fatty acids have implications for the metabolic and pathogenic potential of *K. pneumoniae*. For years, the dogma was that some fatty acids served as carbon sources via degradation through β‐oxidation (Black, 1988; Maloy, Ginsburgh, Simons, & Nunn, 1981). Seminal studies with *E. coli* defined the membrane machinery involved in the uptake and utilization of fatty acids (Black, 1988; Maloy et al., 1981; Nunn & Simons, 1978), a strategy presumed to be linked exclusively to carbon acquisition and degradation through β‐oxidation. Then, inner membrane acyltransferases were discovered to participate in recycling of fatty acids (Zhang & Rock, 2008a, 2008b). Interestingly, there is variable conservation of acyltransferases among bacteria, perhaps alluding to their specific evolutionary development in environmental niches characterized by the presence of specific fatty acids (Zhang & Rock, 2008a). Our thin‐layer chromatography coupled with UPLC/ESI‐MS have added *K. pneumoniae* to the list of Gram‐negatives with the ability to assimilate exogenous fatty acids into their phospholipids.

A bioinformatics survey of sequenced *Klebsiella* species revealed a collection of putative homologs linked to exogenous fatty acid handling. To search for putative homologs, two databases were consulted (Basic Local Alignment Search Tool at NCBI [https://blast.ncbi.nlm.nih.gov/Blast.cgi] and Integrated Microbial Genomes [https://img.jgi.doe.gov/cgi-bin/m/main.cgi] using *Escherichia coli* MG1655 FadL, FadD, PlsB, PlsC, PlsX and PlsY as query sequences and gene product names. The databases revealed numerous homologues to fatty acid transporters, acyl‐CoA ligases and acyltransferases in the selected *Klebsiella* genomes. The recognition of diverse fatty acid structures is presumed to require several homologs of FadL, FadD, and acyltransferases. Indeed, bioinformatic analyses of several *Klebsiella* genomes identified a surplus of homologs predicted to participate in fatty acid uptake and assimilation, including the 2‐acylglycerophosphoethanolamine acyl transferase/acyl carrier protein synthetase (aas). The conservation of this fatty acid handling machinery draws attention to the importance of fatty acid scavenging in the environmental reservoirs of *Klebsiella* species considering their ability to incorporate exogenous fatty acids into membrane phospholipids. The sole carbon experiment may suggest that the fatty acid uptake machinery in *K. pneumoniae* recognizes 18‐carbon fatty acids better than longer chain fatty acids (Figure 1c). A lower level of unsaturation also seems more optimal for utilization as a carbon source.

The observed permeability effects cannot be fully explained. The exogenous fatty acids administered in this study were *cis* isomers. Incorporation of the *cis* conformation would be expected to disturb membrane dynamics, increasing membrane permeability. Interestingly, the position of the first double bond in the fatty acids tested may have affected a trend. For example, the fatty acids that least influenced membrane permeability were linoleic acid (18:2) and α‐linolenic acid (18:3α), whose first unsaturations are located at the more distal carbon position 9. Significant effects on membrane permeability were observed when the first double bond was located at position 6 or lower. Thus, a more kinked fatty acid conformation elicited the greatest effects and, as expected, incorporation of more highly unsaturated fatty acids increased permeability to crystal violet; however, the decreased permeability with eicosapentaenoic acid (20:5) was confounding. Possible explanations for this observation include the activity of enzymes that mediate bacterial membrane homeostasis (Zhang & Rock, 2008a, 2008b). Homology searches using the Basic Local Alignment Search Tool (BLAST) yielded no significant similarity when the fatty acid cis/trans isomerase from *Pseudomonas aeruginosa* PAO1 (PA1846) was used as the inquiry sequence against *Klebsiella pneumoniae* (taxid:573). In the absence of a cis/trans isomerase, desaturases, and cyclopropane fatty acyl‐phospholipid synthases are implicated as the potential modifiers of assimilated unsaturated fatty acids. Nevertheless, the two permeability assays performed support fatty acid‐induced alterations to *K. pneumoniae* membrane permeability.

Since several fatty acids diminished the MICs of polymyxin B and colistin, it is tempting to consider the therapeutic potential of PUFAs during infection, especially considering the emergence of increased colistin and polymyxin resistance among *Klebsiella* strains (Gales, Jones, & Sader, 2011). The results show that presence of several individual PUFAs lowers the MIC for cationic antimicrobial peptides significantly. The importance of Lipid A to AMP resistance is well‐documented (Jeannot, Bolard, & Plesiat, 2017; Olaitan, Morand, & Rolain, 2015; Velkov et al., 2013) but the contributions of phospholipid fatty acyl constitution are largely unknown. Notably, the effectiveness of membrane‐active agents against Gram‐negative pathogens has reinforced bacterial membranes as prime targets for therapeutic attack (Uppu et al., 2017). We interpreted the MIC results as significant for those fatty acids that altered the MIC by at least fourfold as compared with the control. Our results expose another potential vulnerability in bacterial membranes by virtue of exogenous fatty acid remodeling of membrane phospholipids. The mechanism of action appears to be important for fatty acid‐mediated antibiotic resistance since PUFAs did not change sensitivity to the β‐lactam antibiotic imipenem. The incorporation of exogenous fatty acids and subsequent membrane destabilization may facilitate penetration of the antimicrobial peptides, whereas β‐lactamase activity is unaffected by the altered membrane constitution. This strategy may also thwart strains that have developed resistance to triclosan (Curiao et al., 2015), whose mechanism of action targets de novo fatty acid synthesis (Heath et al., 1999). The MICs observed in the current study are higher than previously published (Elemam, Rahimian, & Doymaz, 2010; Lat et al., 2011), particularly with colistin. This may be due to performing the assay in minimal media, since chemically defined media has been linked to higher MIC values (Choi, Chakraborty, Liu, Gellman, & Weisshaar, 2014; Cruz et al., 2013). The only corollary between membrane permeability and antimicrobial peptide susceptibility was that the two fatty acids that elicited significant decreases in permeability also effectively lowered the MICs to both AMPs. Another unknown contributing factor is the differential passage of crystal violet through the outer and inner membranes, which could exaggerate the perceived effect on permeability.

Biofilm formation is an important characteristic of *Klebsiella* strains that are successful pathogens and exhibit multi‐drug resistance (Anes, Hurley, Martinsd, & Fanning, 2017; Vuotto et al., 2017). Our results identified four fatty acids that significantly altered biofilm formation. An elevated production of biofilm was observed when casamino acids were included in the media. While linoleic acid and dihomo‐gamma‐linolenic acid lowered biofilm formation, arachidonic acid and docosahexaenoic acid significantly increased biofilm formation. Our findings are in agreement with Magesh et al. (2013), who observed the inhibitory effect of linoleic acid on biofilm formation. The arachidonic acid‐induced rise in biofilm production is intriguing considering the likelihood of encountering this fatty acid in vivo (Motta et al., 2014). Indeed, bacterial interception of free arachidonic acid would interfere with eicosanoid synthesis and subsequent inflammatory response to infection (Bailie et al., 1996; Ricciotti & FitzGerald, 2011).

Our laboratory and others have contributed to an influx of studies reporting additional impacts of fatty acids on bacterial physiology and behavior, including induction of phenotypic responses involving motility, biofilm formation and virulence (Golubeva et al., 2016; Lai et al., 2005; Liaw et al., 2004; Magesh et al., 2013; Shleeva et al., 2013). Recent studies with *Vibrio* species and *Acinetobacter baumannii* have strengthened the links between exogenous fatty acids and bacterial awareness of these environmental cues (Eder et al., 2017; Moravec et al., 2017). Taken together, our findings in the current study recognize *K. pneumoniae* as capable of assimilating exogenous fatty acids into its membrane phospholipids, as well as fatty acid effects on several phenotypes important for survival and virulence. The potential benefits of PUFAs for *K. pneumoniae* infection have been demonstrated in mice (Sharma, Chhibber, Mohan, & Sharma, 2013). It is hoped that subsequent experiments may further expose vulnerabilities linked to PUFAs that can be exploited for the control and treatment of *K. pneumoniae* infections.

## CONFLICTS OF INTEREST

The authors state that there are no conflicts of interest.
