# Supplementary material for: Exogenous fatty acids alter phospholipid composition, membrane permeability, capacity for biofilm formation, and antimicrobial peptide susceptibility in Klebsiella pneumoniae
Source: Microbiologyopen. 2018 Apr 27;8(2):e00635. doi: 10.1002/mbo3.635 (PMC6391273; doi:10.1002/mbo3.635)

Figure S1. Ultra performance liquid chromatography-mass spectrometry of isolated phospholipids from *Klebsiella pneumoniae* ATCC13883 grown in the presence and absence of fatty acids. Comparison of other fatty acids tested that are not shown in Fig. 3a (main text). All fatty acids tested showed altered chromatograms compared to the control.

Figure S2. Mass spectrometry of individual phospholipids from *Klebsiella pneumoniae* grown in the presence and absence of fatty acids. The parent ion *m/z* indicated is the value that was searched in the LIPID MAPS database (http://www.lipidmaps.org/). Identity of the specific phospholipid is indicated. Note that RT = retention time corresponding to the XIC in Fig 3b. All other fatty acids tested that are not shown in this figure also resulted in similar phospholipid tail alterations corresponding to phospholipid species comprised of at least one acyl chain matching the supplied fatty acid.

Supplemental Figure 1.

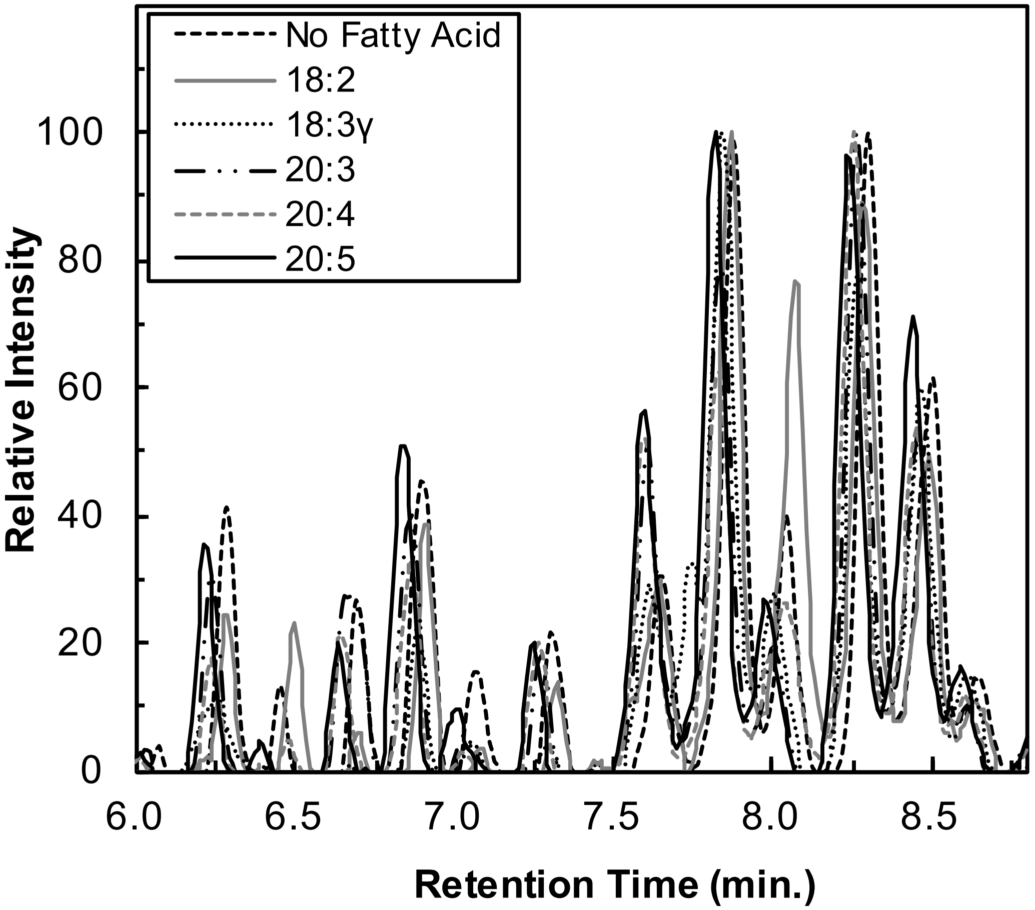

Supplemental Figure S2.

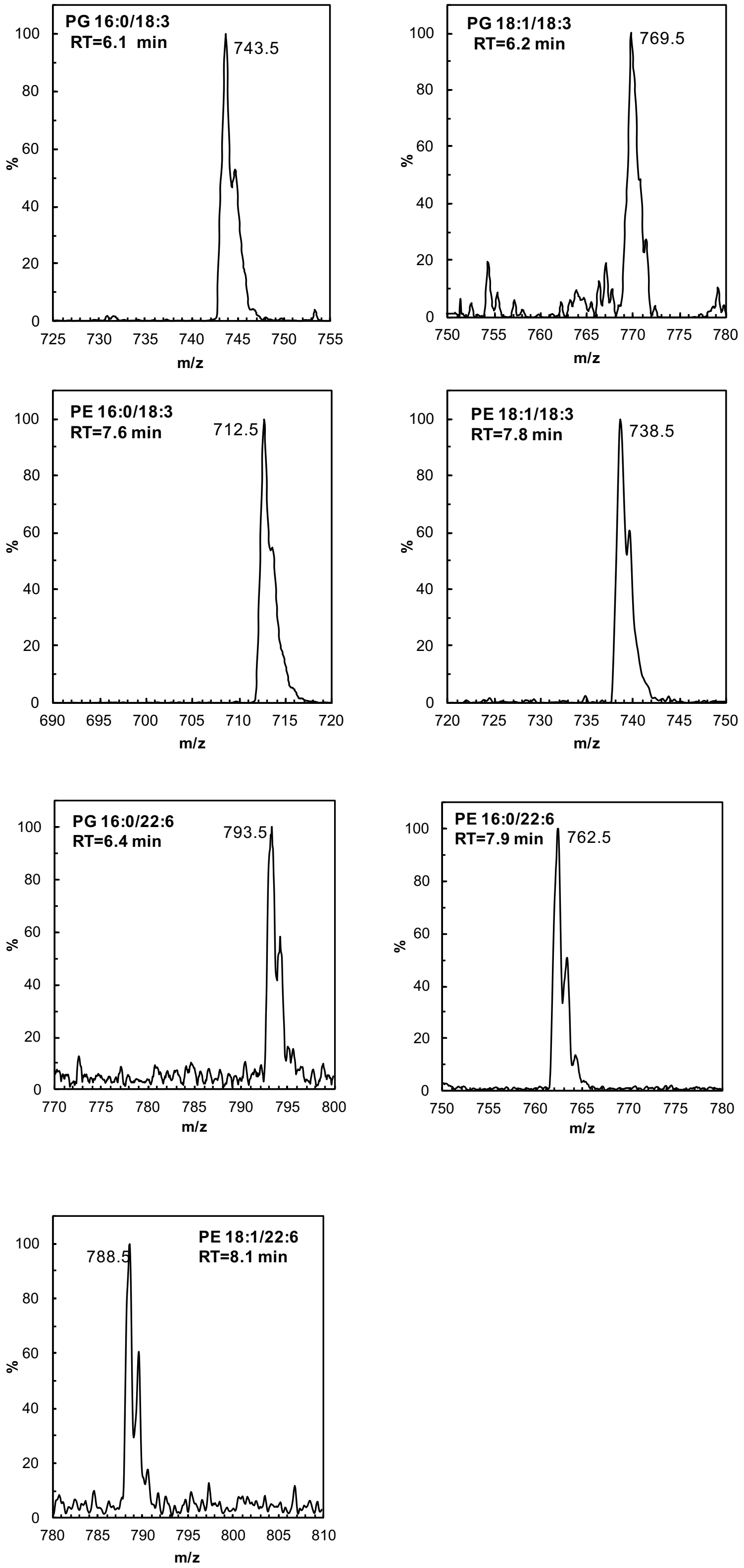

Supplement: Supplementary file 1 [file MBO3-8-e00635-s001.pdf]
